# A fluctuating intensity of care: A qualitative study on the experiences of informal caregivers of patients with critical limb-threatening ischemia

**DOI:** 10.1371/journal.pone.0298959

**Published:** 2024-05-13

**Authors:** Rebecca N. F. G. van Gorkom, Anne L. Meulenbroek, Jolanda de Vries, Donna M. Frost, Lijckle van der Laan

**Affiliations:** 1 Department of Surgery, Amphia Hospital, Breda, The Netherlands; 2 Admiraal de Ruyter Hospital, Goes, The Netherlands; 3 Department of Medical and Clinical Psychology, Tilburg University, Tilburg, The Netherlands; 4 School of People & Health Studies, Fontys University of Applied Sciences, Tilburg, The Netherlands; 5 Department of Cardiovascular Science, University Hospitals Leuven, Leuven, Belgium; University of Verona, ITALY

## Abstract

Informal caregivers play a significant role in providing care for older, often vulnerable, patients, and supporting them as they live with chronic diseases. Due to the rising prevalence of older vascular patients and their use of healthcare, the role of their informal caregivers will become more important. However, little is known about the experiences of informal caregivers of patients with critical limb-threatening ischemia and the impact of informal care on different aspects of these caregivers’ lives. In addition, literature does not describe the burden this role brings with it, or lack thereof. Therefore a qualitative study using a phenomenological approach, specifically interpretive phenomenological analysis, was used to gain insight into the experiences of the primary informal caregivers of patients with chronic limb-threatening ischemia. Data were collected via semi-structured interviews and focus groups discussions. Fifteen primary informal caregivers of patients with critical limb-threatening ischemia under the care of the vascular surgeon at a tertiary teaching hospital in the Netherlands were included. Data analysis yielded three themes: the perceived identity of this group of caregivers; the varying intensity of informal care; and the collaboration between informal carers, their care recipients and the professional care provider within the vascular surgery department. In contrast to carers of other chronic diseases, the shifting intensity of care that informal caregivers of critical limb-threatening ischemia patients experience seems to prevent long-term overload. Adapting to that fluctuating situation requires flexibility from healthcare providers within the vascular surgery department. In addition, professionals need to involve informal caregivers in the patient’s decision-making process and recognize their role in that process.

## Introduction

In a society where the number of chronically ill and older patients is rapidly increasing, informal caregivers play an important role in providing care additional to that given by health care professionals [1]. This is particularly the case when care needs are complex. Critical limb-threatening ischemia (CLTI) is the end stage of a chronic illness that causes ischemic pain and necrosis or gangrene, with the risk of tissue loss in the lower extremities: frailty and a reduction of the quality of life (QOL) are common [2,3]. Treatment aims for limb preservation through revascularization by either open surgery or endovascular interventions. Conservative treatment, consisting primarily of wound care and pain management, is also a viable option for this fragile group of patients. In the absence of these treatment possibilities amputation is often required [2–4]. It is expected that the prevalence of CLTI will rise because of the aging population, with 50–100 new diagnoses of CLTI per 100.000 people within the Western world [2,3]. This will cause a corresponding rise in the need for informal caregivers. In the Netherlands, for example, about four times the present number of informal caregivers will be needed in 2040 to keep up with the aging of the general population [5].

Approximately 30 percent of the Dutch population provides informal care and 16 percent of this care is long-term (over three months) and extensive (over eight hours per week) [5,6]. Informal care is defined as unpaid care given by anyone besides the professional care provider and in which the primary informal caregiver takes the lead [5,7]. Informal caregivers can be partners, children, other family members or acquaintances, and are more often women than men [5]. They commonly find it difficult to see themselves in this role and to identify themselves as caregivers because they feel that the tasks they perform for the care recipient are part of the role of son, daughter or partner and are therefore not necessarily ‘caregiving’ tasks [5,7]. As well, they are concerned about the impact that describing themselves as a caregiver may have on the self-concept of the chronically ill care recipient [7]. Knowles et al [7] emphasize, however, that healthcare professionals are well positioned to recognize and acknowledge the contribution of informal caregivers and to support them in this role. It can indeed be important to do so: about ten percent of informal caregivers in general experience some form of emotional or physical overload, with older caregivers and those with health issues being more prone to this [5]. However, 80% of informal caregivers also report enjoying the good moments of providing informal care. Sharing in these good moments with the care recipient makes the informal caregivers feel good, and feel good about themselves [5].

Many factors are involved in the decision to provide informal care [8,9] For example, cultural self-identity and expectations [5,10] the feeling of being responsible for a care recipient [5,9] or wishing to delay the moment at which more formal care such as admission to a care facility is needed [7,11]. On the other hand, the recipients’ expectations and needs often, among other things determine the extent of informal care and this can differ with socio-economic situation [12] and per disease [5,8,13–15].

In light of this, it seems likely that the experiences and needs of informal caregivers of patients with CLTI will be to some extent unique. Not much is known, however, about the care tasks undertaken by this group of caregivers, about their particular experiences in providing informal care to patients living with CLTI and about the impact this role has on their own lives. The absence of literature about this specific group of informal caregivers makes it more difficult for healthcare professionals to validate their contribution and experiences and support them appropriately. Increased insight into the lived experience of informal caregivers of patients with CLTI can be used to improve the experience of the informal caregivers and to strengthen their role and position. Hence, this qualitative descriptive study aimed to gain insight into the experiences of informal caregivers of patients living with CLTI under vascular surgeon treatment.

## Participants and methods

### Design

A qualitative design with an interpretative phenomenological analysis approach was used, as this approach is suitable for investigating the essence of the experiences [16,17] surrounding the concept of informal care. Both individual interviews and focus group discussions were used, the first to collect in-depth data and the second to explore the full breadth of experiences with informal care [18]. Supplementing the individual interviews more traditionally used in phenomenological approaches with focus group discussions was aimed at utilising the dynamics which focus groups can provide, creating room to elucidate and explore the differences in the experiences of informal caregivers from their own perspectives. There was an intention to highlight areas of consensus and dissension, and to explore differences which arose, for example, from the differing relations of informal caregivers to their recipients of care. Creating room for discussion among the participants was aimed at coming as close as possible to the essential aspects of the experience of being an informal caregiver for patients living with CLTI. Evers [18] argues that the sum total of information generated in this way is more than it would be with individual interviews alone.

### Participants and setting

Patients with CLTI being treated at a tertiary teaching hospital in the south of the Netherlands and their informal caregivers were informed about the study and approached for written informed consent. After consenting to participation in the study, participants were called by the first author to establish whether they met the inclusion criteria. Informal caregivers were included if 1) they were primary informal caregivers of care recipients diagnosed with CLTI; 2) they could convey their experiences; 3) they were older than eighteen; 4) the care recipient did not have a major amputation of the ischemic foot; 5) the care recipient was alive at the moment of invitation, and; 6) the care recipient was being treated at the aforementioned hospital. There were initially no exclusion criteria.

A heterogeneous sample of primary informal caregivers was sought via purposive sampling to ensure rich data was achieved, including adult children, spouses, and de facto partners of the care recipients. Sampling for the individual interviews occurred from October to December 2020 and for the focus group in October and November 2021. One exclusion criteria was added at this time: participants who had been included in individual interviews were excluded from participation in the focus group discussions.

### Data collection

The first author of this article conducted all individual interviews and moderated the focus group discussions; the second author served as a secondary moderator to oversee the group’s interaction and ensure that sufficiently explorative questions were asked. All the interviews were semi-structured to ensure rich data in both known and novel areas [16].

November 2020The seven individual interviews took place between November 2020 and February 2021. All but one were held in a quiet office at the hospital. Because of COVID-19 restrictions, the last individual interview was conducted by telephone. An interview guide based on two starting questions and multiple follow-up questions gave minimal structure to the individual interviews. The starting questions were intended to clarify that we were interested in the experience of the participant themselves as opposed to the experience of their recipients of care. The interview guide hereafter was designed to give participants the space to explore the full breadth of the experience of being an informal caregiver [18,19] as shown in Table 1. A list of topics to prompt discussion in the interviews of all the themes that arose from the literature [18] is also shown in Table 1. The participant being interviewed via telephone filled in a questionnaire before the interview, formed from the topics arising from the literature, to prompt reflection and help enable the same rich data collection as with the face-to-face interviews. Data were collected until no new themes arose, suggesting data saturation had been achieved.

**Table 1:** Individual interview guide.

**Starting questions** **1.**	How are the care recipient’s physical restraints, due to CLTI, affecting you?
**2.**	How does interacting with the doctors and nurses within the vascular surgery department affect you?
**Follow up questions** **Topics**	Do you have an example? How does it make you feel? How do you cope? What does that mean to you? Can you tell me more about that? What does it bring you? Identity Caregiving tasks Motivation Coping Support Relation with care recipient

The two focus group discussions took place in November 2021, when Covid-19 restrictions had lifted enough to permit it. Interpretive phenomenological analysis [17] of the data from the individual interviews had given rise to three themes. The focus group- discussions were structured [18] with one question per theme as presented in Table 2. Beginning broadly and then focusing the discussion was used to obtain both general views and specific ideas from participants [16]. In addition, keywords on a flipchart visible to the focus group participants were used as a summary of the two focus group discussions.

**Table 2:** Interview guide focus-group discussion per theme and sub-topic.

Theme	Sub-topic	Question
1 Informal caregiving as part of one’s identity	1.1 Identity 1.2 Caregiving tasks and motivation 1.3 Boundaries: to uphold or not to uphold	Do you see yourself as an informal caregiver? What kind of support do you provide? 1.3 Why do you give care to your recipient?
2 Varying intensity of care	2.1 Moving along a variable course 2.2 Support: carrying capacity or load 2.3 Handling informal care	2.1 How does providing informal care affect you? 2.2 How do you handle the support you give? 2.3 How does the support you give affect your relationship with the recipient?
3 Why make it difficult when you can do it together?	3.1 Through thick and thin: relating to each other 3.2 Like the tides: the relationship between informal and professional caregivers	3.1 How does the support you give affect your relationship with the recipient? 3.2 What expectations do you have of the healthcare professional within the vascular surgery department?

All interviews were recorded with an audio recorder and transcribed verbatim by the first author of this article.

### Data analysis

The analysis followed the process of interpretive phenomenological analysis in which patterns were sought and identified that were related to the research question. The process of analysis was iterative, occurred concurrently with data collection and was based on the intended meaning rather than the frequency of occurrence [16,17]. By doing so, themes and sub-topics were created while comparing codes and units of meaning within and across interviews and focus group discussions. Atlas.Ti software (Version 9.0.22.0 15-12-2021) was used during the analyzing process [20].

### Validity and reliability

This study followed the criteria written up by Cope [21], Smith et al [16], and Zahavi [22], to ensure the validity and reliability of this study. A test interview was conducted in preparation for the study to improve the skills of the interviewer. Her interview skills were analyzed and discussed in hindsight with the support of a supervisor. This method was used to improve the quality of each next interview or focus group discussion. Using the flipchart to summarise the content of the discussion during the focus-group interviews enabled participants to review, revise and confirm the record being made. The analysis of all the interviews was peer-reviewed by two peers. Keeping detailed notes contributed to an accurate data trail and supported reflexivity and bracketing of assumptions [22].

### Ethical considerations

This study did not require approval by the Dutch Medical research involving human subjects act and was performed in conformity with the Declaration of Helsinki [23]. It was registered in Castor study management software and approved by the board of directors of Amphia Hospital under registration number N2020-0368 [24]. Confidentiality for the participants was maintained by the use of their affiliated patient’s patient number instead of their name, and via anonymisation of the transcripts prior to peer review of the analysis. The voice recordings of the interviews were only accessed by two of the authors of this article (the interviewer and one of the peer reviewers). These procedures were described in the information letter provided to participants as part of the informed consent process, and were agreed to by those who gave consent.

## Results

**Fig 1:**
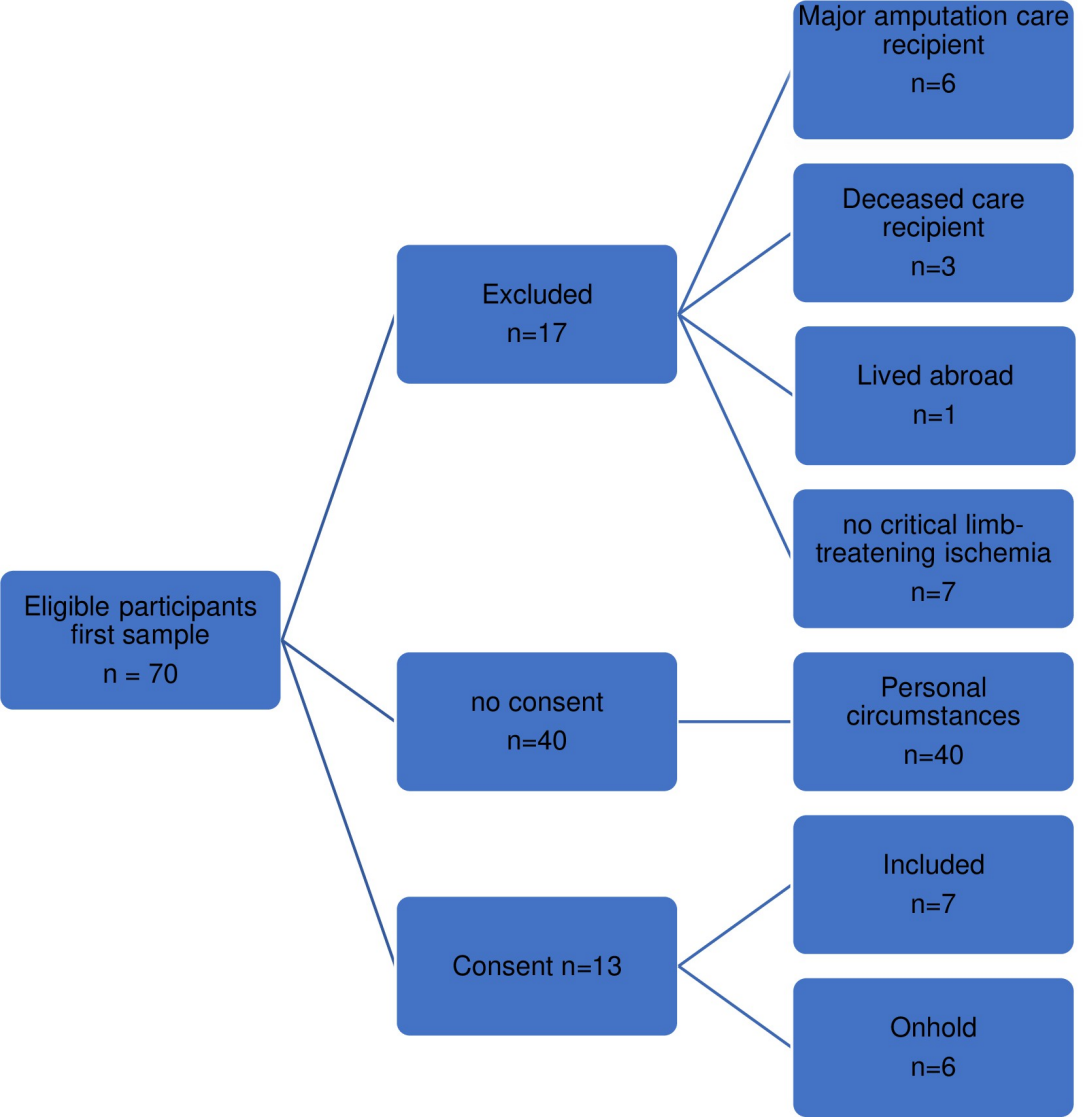
In S3 Flowchart first sample.

Fifteen of the 90 eligible informal caregivers of patients with CLTI participated in this study. The sampling process is summarized in Figs 1 (individual interviews) and 2 (focus group interviews).

**Fig 2:**
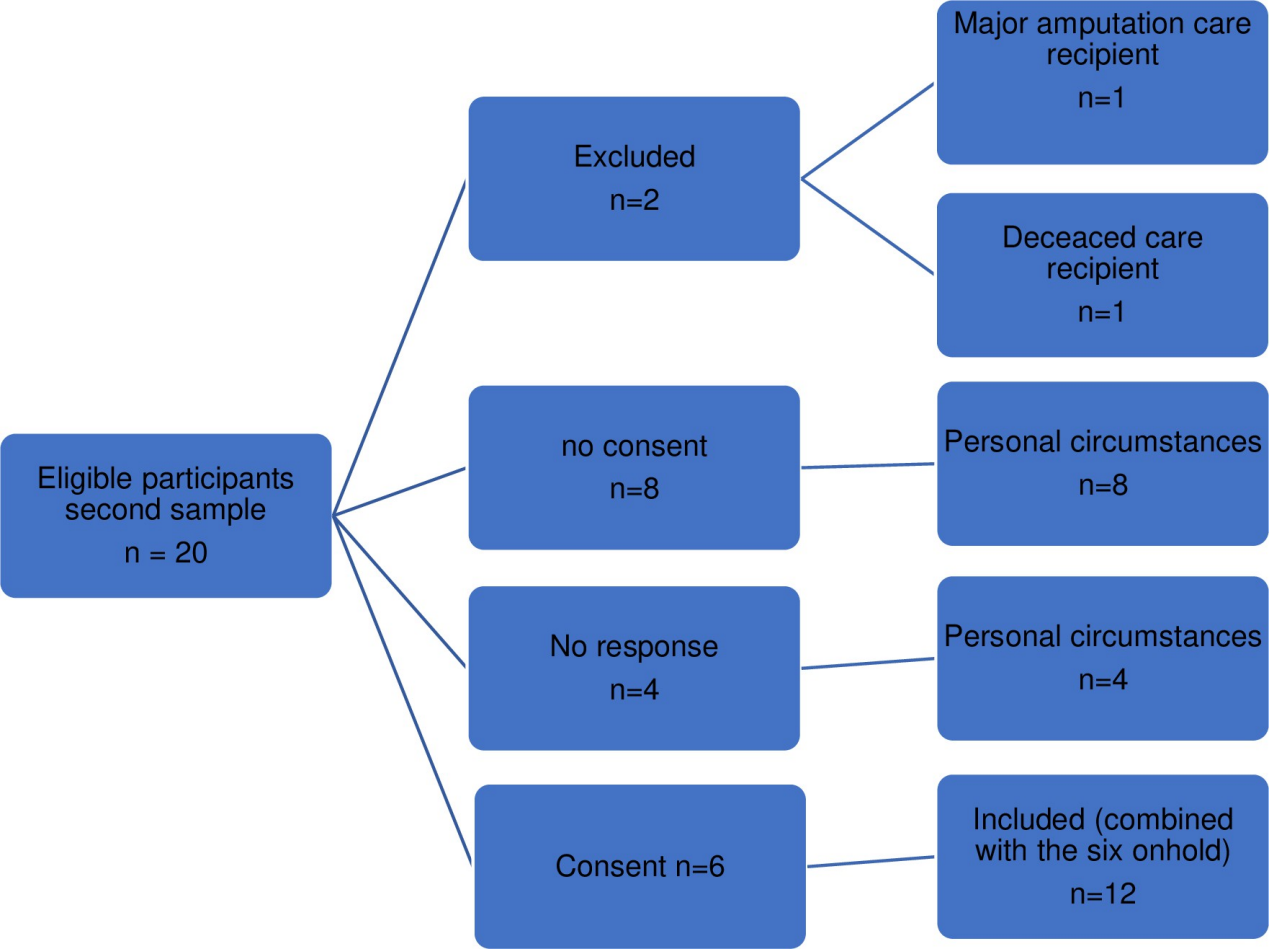
In S4 Flowchart second sample.

As shown in Table 3, the participants formed a group with varied individual characteristics yet homogeneous in that they all provided long-term and extensive informal care [5] for patients living with CLTI.

**Table 3:** Baseline characteristics of the participants (n = 15).

Sex Male Female	7 8
Age (years) 40–50 50–60 60–70 70–80	1 5 2 7
Relationship to care recipient Husband Wife De facto partner male De facto partner female Daughter Son	4 3 2 1 4 1
Duration of caregiving (years) 1–2 3–5 6–10 11–20 > 20	4 5 2 2 2
Frequency of caregiving (days per week) 1–2 day 7 days	3 12
Living in with care recipient Yes No Sharing informal care Yes No	10 5 6 9
Employment informal caregiver Pension Unemployed Fulltime own boss 0,5–1 day per week 3–4 days per week	5 4 1 1 4
Treatment course CLTI Revascularization operation Percutaneous Transluminal Angiography (PTA) Revascularization operation and PTA Conservative	3 6 3 3
Comorbidity care recipient Yes No	15 0
Health issues of the care recipient Cardiac Pulmonary Renal Diabetes Malignancy Neurocognitive Others	13 1 3 9 3 6 6

The care recipients receiving care from the participating informal caregivers had undergone various treatments, for example, operations, angioplasty, or conservative treatment. The latter included lifestyle advice, pain management, and wound care. All 15 participants cared for recipients who suffered from comorbidities like cardiac or renal problems, diabetes, or neurocognitive problems. Furthermore, the period of caregiving often exceeded the presence of CLTI because of the comorbidities that recipients already struggled with at the time of the diagnosis of CLTI. After inclusion, the care recipient of one of the informal caregivers who participated in this study passed away. After careful consideration, the participant decided to take part in the focus group discussion.

In the course of this study, three themes were identified that gave insight into the experience of the informal caregivers for care recipients living with CLTI: 1) informal caregiving as part of one’s identity; 2) varying intensity of care; 3) why make caring difficult if you can do it together? Each of the themes contains sub-themes, as described below.

### Informal caregiving as part of one’s identity

Caregiving tasks, management of boundaries, and personal activities affect the perceived identity of informal caregivers. However, the perceived identity of the informal caregiver also seemed to affect the importance they placed on the caregiving role and the manner in which they fulfilled it.

### What’s in a name?

All participants knew of the term ‘informal caregiver’. The majority, eleven of the fifteen participants did not use this term to describe themselves. A single participant found the expression too formal to express what her role consisted of as an informal caregiver and the majority found the caregiving tasks performed to be their responsibility as a partner or adult child. Two of the participants did identify with the term informal caregiver. When explicitly describing themselves as ‘informal caregivers’, caregivers reported more easily accepting help from their surroundings and being able to access formal support from community organizations such as peer support and financial contributions from the municipality.

*A year or two ago they told me that I should sign up as an informal caregiver to get financial compensation from the municipality*. *But I said*, *I only take care of my husband*. *I do not think that this compensation is for me*! *(Individual interview*, *spouse of care recipient*.)

Furthermore, factors such as hobbies, work or voluntary work, and social contacts were important to the perceived identity of all participants. It was reported that these aspects of their lives were necessary to enable them to feel like they were part of a larger picture and that they were contributing to society. When such activities had to be curtailed it had a negative impact on the self-identity and feeling of self-worth of the informal caregiver.

*And then I stopped all my hobbies and stuff*. *Yeah*, *I had quite a bit of trouble with that*. *Because uhm*, *yeah*, *suddenly you’re nobody*. *It may sound a bit crazy*, *but I was once someone myself*. *I am [now] out of the working society and do not work as a volunteer anymore*. *[Yet] these things made me feel very useful to society*. *(Individual interview*, *spouse of care recipient*.)

Whether or not participants identified themselves as ‘informal caregivers’, they saw providing care for the care recipient in their lives as integral to their own perceived identity.

### Duty of care

All participants engaged in various caregiving tasks involving practical, physical, and emotional support and guidance for the care recipient. As an internal motivation for informal care, all participants who cared for a parent stated they owed it to the parent to take care of them because the parent had been there for them. All ten partners of care recipients explained that they had vowed to support their partner for better and for worse. Among these participants unwedded partners stated the same responsibility towards their care recipients as wedded partners did. This aspect of the experience of informal caregiving includes a hesitation to ask for help due to not wanting to be a burden to others.

*“As long as I can do it myself I will*. *My children live further away*, *work and have their own family*. *I do not want to burden others*.*” (Individual interview*, *spouse of care recipient*).

Where help is not offered, the caregiving tasks are taken up by the informal caregivers themselves without real consideration for their own burden. The motivation to continue providing informal care can be intensified by the fear of losing a partner and care recipient due to admittance to a long term care facility after an inpatient stay on a vascular surgery ward, for example during the Covid-19 pandemic. In these instances the care recipients returned home after discharge from hospital and the informal caregiver cared for them themselves.

*The hospital had asked for home care but there was none*, *so for me*, *the choice was to take my wife to a nursing home or to do it myself*. *I do not want to send my wife to a nursing home*, *especially during Covid-19*, *because then I will never see her again*. *(Individual interview*, *spouse of care recipient*.)

### Crossing lines and shifting boundaries

All participants expressed crossing personal boundaries and perhaps having to shift them as a feature of being an informal caregiver for patients living with CLTI. When the caregiver has a live-in relationship with their care recipient this pushing at boundaries feels inevitable. Crossed boundaries may be the result of external factors like the limitations of healthcare services in combination with the needs and wishes of the care recipients, and the urgency created when the health status of a care recipient deteriorates. Boundaries can shift, perhaps temporarily, because of the varying intensity of the health status and treatment course of the care recipient. When boundaries are crossed, or shifted, particularly when the caregiver had previously attempted to draw a line that would not be crossed, the burden on the informal caregiver tends to increase temporarily.

*I kept pushing those boundaries*. *I had decided I would never do physical care for my parents because I lacked the training and I would not know how*. *But last week my mother had antibiotics and she got diarrhea from that*. *Well*, *I was there and those pants were dirty*, *I could not leave her in them either*. *(Focus group*, *daughter of care recipient*.)

The moment of crossing a boundary doesn’t always feel like a choice. It can cost more energy however to draw a line and maintain it and participants were aware of their boundaries shifting, sometimes repeatedly.

### Varying intensity of care

Dealing with the variable course of caring for a patient with CLTI results in a fluctuating experience of one’s own carrying capacity and load. It can involve a fear of the future and, for these participants, the varying intensity of care was felt particularly during the covid-19 pandemic.

### Moving along a variable course

Providing informal care for patients living with CLTI is characterised by a fluctuating intensity of informal care that has to do with the treatment course, the amount of pain of the recipient, the level of dependency of the recipient, and the recipient’s other health problems as well as the health problems of the informal caregiver. Half of the participants whose recipients had surgery stated that it caused temporarily added stress. On the other hand, the other half stated that surgery can also give the caregiver a sense of relief based on the expected gain for the recipient after the treatment.

*She had surgery again and she was in more pain afterward*, *so that was added misery*. *Luckily that goes away and things are expected to get better*. *(Focus group*, *de facto partner of care recipient*.)

An additional burden is present when daily travel to and from the hospital is needed, and/or spending multiple hours on the ward to accompany and support the care recipient. This takes a toll, especially on married and de facto partners. Adult children caring for a parent seem to benefit more from the additional professional care available during an inpatient admission. The presence of professionals to watch over the care recipients and who take on tasks that the informal caregiver can let go of, for the time being, unburdens those informal caregivers. One informal caregiver who cared for her mother explained that she did experience less of a burden during the admittance of the recipient. In such situations informal caregivers may note, however, an increase in things to arrange, like visits and transportation at discharge. When communication between healthcare facilities on transfer is not effective it creates an added burden which can persevere after discharge from the hospital. In contrast, when this aspect of formal care goes well, informal caregivers feel satisfied with the caregivers of the vascular surgery department, are relieved of having to themselves communicate medical information about their care recipient to another healthcare organisation and may consider the effective communication to contribute positively to the level of care the recipient receives.

*What I have recently experienced*, *due to you*, *is that a letter then goes from your [hospital] department to the medical department of [other healthcare provider] and that letter does do its job*. *That was very striking because I suddenly said to my husband*, *‘Look at this*, *nothing has happened for 2 weeks*, *even if I ask about it*, *especially for a physio*, *dietician*, *you name it*. *And that letter comes in*, *and the next day there is a whole report and I think hey*, *something has been sent and now we will get a response*. *And I like that very much*. *(Focus group*, *daugther of care recipient*.)

### Fear for the future

Hospital admission of the care recipient can cause an increase in worry about their health situation for the informal caregiver and two participants described worrying more and more about the health situation of their care recipients. This was particularly the case when the care recipient has already endured amputation of one or more toes and/or multiple revascularization attempts via operation or angioplasty. In these circumstances the stress is increased by the fear of amputation of a leg and the possible disability as a consequence of that, or even death.

*It is the fear I have because I just do not know where it will end after the amputation of several toes*. *It starts with a toe but yeah*. *I think that it is not going to be the end of that story*. *(Focus group*, *de facto partner of care recipient*.)

Further, the health status of the informal caregiver was identified as a possible burden that could also fluctuate in intensity. Two participants who cared for a partner were afraid that they would no longer be able to care for their recipients due to the deterioration of their own health in the future.

*I am afraid of a time coming that I cannot do it anymore*. *If my wife is still here*, *she cannot go on without my help*. *That is one of my fears*. *She is so dependent and I find that very scary*. *(Individual interview*, *spouse of care recipient*.)

Another aspect of concern about the future is the fear, or even dread, as stated by one of the participating partners, that the care recipient’s CLTI related problems would last a lifetime.

### Dealing with the Covid 19 pandemic

Most participants described that the Covid-19 restrictions highlighted the flexibility required of informal caregivers in their role as they were faced with challenges which changed the intensity of care in unforeseen ways. The restrictions limited the ability of informal caregivers to accompany care recipients to outpatient appointments, for example, and to visit during inpatient stays. All participants stated to understand the circumstances but some found it challenging to stay involved in the treatment process of their care recipient in the manner they preferred. At the same time, they were keen to protect their care recipients, who they recognised to be in fragile health, from the risk of infection with Covid-19. This meant that people who did not comply with the restrictions could be seen as possible threats to both the health of care recipients and to moving towards a lessening of the need for the restrictions. Limiting time spent outside of the house in this period, to reduce the risk of infecting their care recipient, was one way informal caregivers could manage this perceived threat.

*I noticed that I am very careful because I know how vulnerable my wife is*. *I wash my hands frequently and I try to be as careful as possible*. *And I just do not go anywhere right now except for groceries*. *(Individual interview*, *spouse of care recipient*.)

On the other hand, one participant stated that the periods of lockdown during the pandemic lightened the caregiving load, in some ways. There was more time available to spend with care recipients, for example, and care recipients themselves had less social activities, reducing their need for assistance from the caregiver. This could engender a sense of relief in the informal caregiver. On the other hand, having to give up personal activities could also make the caregiver’s world smaller, limiting those activities which contributed to self-care and helped the caregiver to be able to care for the care recipient daily.

*After I get home at about noon [outside of the lockdown]*, *I am there for the whole day but I’ve already had my time*. *That fell away during the pandemic*. *I have noticed it is important to me*. *I can only be a good caregiver if I take care of myself*. *On the other hand*, *I have more time at home to spend with my husband*. *(Focus group*, *spouse of the care recipient*.)

### Support from others: Help or hindrance

For all participants, a flexible environment was needed to maintain the balance between informal care and other life tasks. Especially for four of the five participants that cared for a parent while having jobs and families of their own.

*I work at home so I can do that whenever I want*. *If I have to go to an appointment during the day*, *I do my work in the evening*. *It makes it easier to organize*. *Also*, *my kids are grown enough so that they do not need me all the time*. *This gives me a lot of freedom to take care of my dad*. *(Individual interview*, *daughter of care recipient*.)

Of the fifteen participants, eleven accepted help from their surroundings and experienced it as a relief from the burden of informal care. But the involvement of others can be experienced as interfering and burdensome, particularly if accompanied by negative comments. Outside help that interferes with the habits and routines of the caregiver can feel like losing control.

For example, when home care professionals come into the house to take care of the care recipient. Dependency of the care recipient on these home care professionals can create an extra burden for the informal caregiver, even when it is the partner that depends on the home care. Furthermore, three participants had experiences with peer support groups which could either be positive or increase the stress of the situation. One of them saw it as a way to help her carry the load of care.

*I had a meeting with my peers and I told them that I was faced with a dilemma*. *I told my husband he could not have a cigarette*. *Then someone asked me how old my husband is*. *He said*, *what nonsense*. *Let him think for himself*, *you are already having a hard time*. *Are you going to fight that cigarette now too*? *(Focus group*, *spouse of care recipient*.)

The other two informal caregivers had the opposite experience and described the encounters with peer support groups as unhelpful and even unpleasant, for example because of the pedantic tone during the meeting. One informal caregiver assumed that his care recipient would not agree to having her situation discussed in such a setting, and this prevented him from seeking peer support.

Additionally, all but three informal caregivers describe acquiring professional support and navigating their way through the possibilities to be difficult and a largely unknown path, due to all the regulations and the ever-changing landscape of care systems and organisations. Furthermore, the information informal caregivers received during hospital admittance of the care recipient was marginal.

### Coping with informal care

To cope with informal care, all participants adjusted to the situation. Eight out of ten participants that cared for a partner dealt with informal care by using emotional coping strategies such as accepting the situation, as did two of the five participants that cared for a parent. One of the two participants that cared for a parent lived with the recipient and described the recipient as demanding.

*I was not allowed to go for a bike ride because then my mother would be alone*. *So*, *it has been tough lately*. *But I accepted that*. *(Focus group*, *daughter of care recipient*.)

Out of five participants that cared for a parent, three lowered their burden by solving problems through sharing informal care, adjusting their work-life, and stimulating the independence of their care recipients. One of these partners took good care of her own needs in order to be able to provide informal care. The other partner lived separately from his recipient. Two out of ten participants who cared for a partner also adjusted by using technology or shortening their own work week.

*I let my father do things on his terms*. *I support him where necessary and he keeps his independence*, *which I think is important*. *It relieves me in a way because I do not have to jump in every time*. *My brother and sister and I each have a day on which we take care of my father*. *The rest of the time I am busy with my work*, *I dropped a day off to take care of my dad*, *and my family so I have plenty of support*, *and that makes it easier*. *(Focus group*, *son of care recipient*.)

Some strategies are both practical and strengthen emotional coping, such as taking good care of one’s own needs while caring for your partner, or living separately from the partner who is the care recipient.

### Why make caring difficult if you can do it together?

This theme describes the experience of the relationships between the informal caregiver, care recipient, and professional healthcare provider within the vascular surgery department.

### Through thick and thin: Relating to each other

Of the participants that cared for a partner, nine confirmed having to collaborate with the recipient of care to manage CLTI and the effects of the condition, which tended to make the relational bond stronger than it was previously. Additionally, all but one participant stated that the division of roles between partners did not necessarily change much. There can be practical differences between the partners, such as the division of most household tasks. Many partners felt that the gratitude they received from their care recipients made it easier to form that added bond, although such a strengthening of the relationship was not inevitable. In addition, intimacies changed over time.

*It is different*, *I and my wife have not had sex for several years*. *But we are still cuddling*. *So not much has changed in that regard*. *(Individual interview*.)

Of the ten, one participating partner stated that the inequality in health between partners can take its toll on the relationship, causing an imbalance in dependency between them. This imbalance can resolve, for example, when the health of the informal caregiver deteriorates, restoring a sense of equality between the caregiver and the care recipient.

*There was a period when—and that may sound a bit silly—when I was not sick myself*. *Yes*, *it was an unequal situation*, *and now I am a vascular patient myself*. *Now there is a balance between us*, *which makes us more equal*. *(Individual interview*, *spouse of care recipient*.)

Four out of five participants that cared for a parent reported that the role division between child and parent tends to feel reversed with the adult child now being the responsible party. This shift too was not inevitable. In some situations the adult child caregiver regards themselves as more of an equal to the care recipient, as described by one of the participants. This was explained as a consequence of guarding the parent’s autonomy.

*Our relationship has been on a comparable level for a while*, *so no longer father and son but more equal*. *I do not take up tasks he can do for himself*. *I stimulate him to do as much as possible*. *(Focus group*, *son of care recipient*.)

One participant stated that she chooses not to argue with her partner about health-threatening behavior like smoking. In this way she preserved the peace in the house and the choice helped her to remain in her role as a wife, and not become her care recipient’s guardian.

### Like the tides: The relationship between informal and professional caregiver

All fifteen participants wanted to be involved in the decision-making process of their care recipients’ treatments. All but one stated that being able to observe and practice tasks like wound care during an outpatient consultation is helpful in learning how to support the care recipient. Also, receiving information accompanied by drawings or by being shown medical images, such as CT or MRI scans and having them explained, helps informal caregivers to remember the information correctly and to feel secure in performing certain tasks. The one participant that did not receive visual information stated that he did not receive enough information. Not receiving information in an appropriate way can contribute to the informal caregiver not feeling recognized as an important party in the conversation with the vascular surgeon and the recipient of care. Furthermore, twelve participants stated that when doctors, nurses and nurse practitioners speak in a manner which informal caregivers can understand, they feel seen and involved in care. This is not the case when health professionals use more complicated jargon. The use of medical jargon can come across as arrogant.

*I think it shows arrogance*. *We do not know any medical jargon so just tell us like it is without difficult words*. *If I do not understand I will ask for clarification*, *my parents will not do that*. *(Focus group*, *daughter of care recipient*.)

Lack of consistency between different doctors during inpatient admission causes additional stress to an already stressful situation.

*No*, *I do not always think communication is good*. *I would like it to be more clear*. *I often see other doctors at her bedside*. *When the wound did not close*, *a different doctor than our own came and out of the blue said that the foot needed to be amputated*. *It was not the case at all but her doctor had to make up for that*. *It caused us a lot of stress and fear for the worst*. *(Focus group*, *partner of care recipient*.).

Regarding the period after discharge, it is important for informal caregivers to receive information about possible scenarios they may face. In addition, they find it important to be told where to go in the case of questions or problems encountered with the health of their care recipients. Informal caregivers expect this approach to lower the burden in sometimes already stressful occasions and to relieve worry about their care recipient’s healthcare situation.

## Discussion

The findings of this qualitative study reflect the changeable experiences of informal caregivers of patients with CLTI. Acknowledging that the experiences of this specific group of informal caregivers differ in some ways from those of the caregivers of elderly and chronically ill patients means recognizing that their needs can also differ. Acting on this insight can contribute to a constructive relationship between formal and informal caregivers in the care for CLTI patients. This study, then, gives professional healthcare providers within the vascular surgery department the tools to interact with informal caregivers in a way that contributes to supporting their care activities as well as the patient’s disease management process.

The common denominator within the three themes was the varying amount of burden experienced by informal caregivers during the processes patients with CLTI go through. This varying course meant that informal caregivers experienced a fluctuating amount of caregiver burden, instead of a progressive amount, as is often described among elderly and chronically ill patients [1,15].

In the absence of data specifically for informal caregivers of patients with CLTI, findings were compared with studies of elderly and chronically ill patients. Some of those findings were comparable to the results of this study. First of all, informal caregivers that identified with the term seemed to more readily accept outside help [7,25]. Additionally, they too find it hard to see themselves as informal caregivers because of the overlap between informal caregiving and the caregiving tasks they would in any case perform as an adult child or partner [13]. Second, maintaining other life tasks and activities were important to the perceived identity of the informal caregivers and subject to change during the caregiving process. Third, extrinsic factors, like the degree of dependence or the wishes of the care recipient, determined caregiving tasks and boundaries [14,26]. Whereas the opportunity to uphold boundaries added to the wellbeing of the informal caregiver [9] Furthermore, few participants actively engaged in peer support and had a positive experience as confirmed in the literature [5]. Notably, the participants in the focus group discussions experienced the interaction with other informal caregivers during the interview as positive, even though the experience with peers in other situations was at times perceived as unpleasant. Another similarity to current literature is the point that informal caregivers of recipients with CLTI want to be involved in the decision-making process between professional healthcare providers and patients [1,27].

In contrast to informal caregivers of recipients with chronic conditions, who often experience a progressive amount of burden, the varying intensity of care that informal carers of CLTI patients experience, seems to prevent long-term overload [26]. Another contrast to the literature is with respect to the increased burden experienced by informal caregivers during the COVID-19 pandemic [28]. This study shows a little more nuance to that claim. Informal caregivers in this study understood that this temporary situation was necessary to protect their care recipient from infection. In addition, there were also benefits, such as having more time to spend with the care recipient during the lockdown. The third difference is that the type of intimacy changed into another form of affection between the informal caregiver caring for a partner and the recipient of care. This change was not perceived as negative, whereas the current literature describes the loss of intimacy between partners and regards it as an undesirable effect on the relationship [26].

A specific finding of this study was the difference in the way informal caregivers seem to handle the burden of care. Children who cared for their parents more often chose an instrumental approach to solve a problem. Partners seemed to choose an emotional approach in which they tend to accept the situation rather than change it. There were exceptions, such as a daughter who cared for and lived with her mother also choosing an emotional approach. Similarly, the one partner who lived apart from his care recipient seemed to have a more instrumental approach. This detail suggests that not only the type of relationship but also the living situation is an important factor in the chosen coping strategy.

### Practical implications

The findings of this study underline the need for professional caregivers to involve informal caregivers in the treatment process of patients with CLTI under hospital care. Furthermore, because informal caregivers often do not ask for help themselves, professional care providers should actively ask about the need for assistance, information and support. In addition, there should be clear communication between the professional healthcare provider and the patient, but also between the healthcare provider and the informal caregiver. The focus of this communication should be on the perceived caring capacity or burden of the informal caregiver, the coping mechanism that is being used, and the collaborative relationship between the patient, the informal caregiver, and the professional care provider. In doing so, the results of this study show that the individual situation of each informal caregiver and their recipient need to be taken into account. Because of the fluctuating intensity of the treatment process of patients with CLTI, and with that the varying perceived burden of informal caregivers, both the situation and the need for support require frequent assessment. If health professionals pay attention to uncovering this information, the desired and needed intervention can be initiated. Finally, to be able to recommend a suitable intervention, the professional healthcare provider needs to be up to date on the interventions available within and outside the organization.

### Strengths and limitations

The possibility of researchers’ bias was limited by continuing to reflect on the interpretation of the data in collaboration with multiple peers and participants. Nonetheless, this study did have some limitations. Because of the COVID-19 pandemic, it was difficult to recruit enough informal caregivers to participate. In consequence, there were no informal caregivers included outside of the role of partner or child. Additionally, the COVID-19 restrictions on accompanying the care recipient to appointments or to visiting during admittance may have influenced the experience of the caregivers in a manner that does not exist outside of the pandemic. COVID-19 restrictions also meant that the two focus-group interviews each took place with four participants instead of five or six as recommended in the literature [17,18]. However, data saturation was achieved.

Since this study was performed in a single center, it is conceivable that the in-house culture has contributed to the data regarding the interaction between informal caregivers and professional healthcare providers within the vascular surgery department. Because of the absence of informal caregivers besides those in a partner or child relationship with the care recipient [9], and the low number of participants that did not know their recipient before the diagnosis of CLTI, the themes described do not include these perspectives. In future studies, the heterogeneity could usefully be expanded to include a wider range of experience. Furthermore, it would be interesting to investigate if informal caregivers for amputees due to CLTI have different experiences. Lastly, the difference in coping strategy between informal caregivers that do and do not live with their recipients can be further investigated to assess whether the living situation is indeed an indicator of the chosen coping style.

## Conclusion

Overall, this study seems to show that informal caregivers of patients with CLTI experience a less progressive burden than informal caregivers of elderly patients and those with other chronic diseases, which potentially prevents overload. Furthermore, professional healthcare providers within the vascular surgery department need to acknowledge both the role of informal caregivers in the variable course of patients with CLTI under hospital care, and their chosen coping strategy to fulfill their needs. By including informal caregivers in the decision-making process of the patient, and by actively involving them in the communication, informal caregivers feel part of the process and valued for their role by healthcare professionals. In doing so, appropriate support for informal caregivers can be provided.
